# Supplementary material for: Resolving the full spectrum of human genome variation using Linked-Reads
Source: Genome Res. 2019 Apr;29(4):635–45. doi: 10.1101/gr.234443.118 (PMC6442396; doi:10.1101/gr.234443.118)
Supplement: Supplemental Material [file supp_gr.234443.118_Supplemental_longranger-2.2.zip › longranger-2.2.tar.bz2!/lr2.2-public/lib/stan/cmdstan/stan/doc/api/html/math_2functions_2log1m__exp_8hpp_source.html]

Stan: src/stan/math/functions/log1m\_exp.hpp Source File


|  |  |
| --- | --- |
|  | Stan  2.5.0  probability, sampling & optimization |


- Main Page
- Related Pages
- Namespaces
- Classes
- Files

- File List
- File Members

All Classes Namespaces Files Functions Variables Typedefs Enumerations Enumerator Friends Macros Pages

- src
- stan
- math
- functions

log1m\_exp.hpp

Go to the documentation of this file.

1 #ifndef STAN\_\_MATH\_\_FUNCTIONS\_\_LOG1M\_EXP\_HPP

2 #define STAN\_\_MATH\_\_FUNCTIONS\_\_LOG1M\_EXP\_HPP

3

4 #include <boost/math/tools/promotion.hpp>

5 #include <stdexcept>

6 #include <boost/throw\_exception.hpp>

7 #include <boost/math/special\_functions/expm1.hpp>

8 #include <stan/math/functions/log1m.hpp>

9

10 namespace stan {

11  namespace math {

12

38  template <typename T>

39  inline typename boost::math::tools::promote\_args<T>::type

40  log1m\_exp(const T a) {

41  if (a >= 0)

42  return std::numeric\_limits<double>::quiet\_NaN();

43  else if (a > -0.693147)

44  return std::log(-boost::math::expm1(a)); //0.693147 is approximatelly equal to log(2)

45  else

46  return log1m(std::exp(a));

47  }

48

49  }

50 }

51

52 #endif

stan::agrad::expm1

fvar< T > expm1(const fvar< T > &x)

**Definition:** expm1.hpp:15

log1m.hpp

stan::math::log1m\_exp

boost::math::tools::promote\_args< T >::type log1m\_exp(const T a)

Calculates the log of 1 minus the exponential of the specified value without overflow log1m\_exp(x) = ...

**Definition:** log1m\_exp.hpp:40

stan::agrad::log

fvar< T > log(const fvar< T > &x)

**Definition:** log.hpp:15

stan::agrad::exp

fvar< T > exp(const fvar< T > &x)

**Definition:** exp.hpp:16

stan::math::log1m

boost::math::tools::promote\_args< T >::type log1m(T x)

Return the natural logarithm of one minus the specified value.

**Definition:** log1m.hpp:40


---

[ Stan Home Page ]

*© 2011–2014,
Stan Development Team.*
